# Comparison of MTA versus Biodentine in Apexification Procedure for Nonvital Immature First Permanent Molars: A Randomized Clinical Trial

**DOI:** 10.3390/children9030410

**Published:** 2022-03-14

**Authors:** Yasser Alsayed Tolibah, Chaza Kouchaji, Thuraya Lazkani, Ibrahim Ali Ahmad, Ziad D. Baghdadi

**Affiliations:** 1 Department of Pediatric Dentistry, College of Dentistry, Damascus University, Al-Mazzeh St., Damascus P.O. Box 3062, Syria; firedragoon1994@hotmail.com (Y.A.T.); shazako@yahoo.com (C.K.); 2 Department of Restorative Dentistry and Endodontics, College of Dentistry, Damascus University, Al-Mazzeh St., Damascus P.O. Box 3062, Syria; dr.thuraya1979@gmail.com; 3 Dentistry Department, Al-Wakra Hospital, Hamad Medical Corporation, Al-Wakra P.O. Box 82228, Qatar; ibrahimali79@yahoo.com; 4 Department of Preventive Dental Science Division of Pediatric Dentistry, Dr. Gerald Niznick College of Dentistry, University of Manitoba, Winnipeg, MB R3E 0W2, Canada; 5 Toddlers to Teens Dental, Greenwoods Dental and Surgical Centre, Winnipeg, MB R2L 0Y5, Canada

**Keywords:** apexification, apical plugs, Biodentine, MTA, PAI, VAS

## Abstract

This study aimed to evaluate the radiological and clinical outcomes of Biodentine apical plugs compared to mineral trioxide aggregate (MTA) in treating immature molars with apical lesions in children. Materials and Methods: Thirty immature roots of 24 permanent lower first molars with apical lesions were randomly divided into two groups: group 1 (15 roots) treated with MTA apical plugs and group 2 (15 roots) treated with Biodentine apical plugs. Treatment radiological outcomes were assessed using the periapical index (PAI) scale after 6 and 12 months of treatment. The presence or absence of apical calcified barrier (ACB) was assessed after 12 months of treatment. The visual analog scale (VAS) was used to compare the postoperative pain between the two groups after 1, 3, 7, and 14 days of treatment. PAI scores between the two groups were compared using the Mann–Whitney U test, the presence or absence of the ACB was compared using the chi-square test, and the VAS scores were compared using the *t*-test. The statistical significance threshold was set at 0.05. Results: There were no statistically significant differences in the PAI between the two groups at 6 and 12 months postoperatively. After 12 months, four cases in the Biodentine group showed ACB formation, whereas ACB was not found in any case treated with MTA. The VAS scores were statistically lower in the MTA group on the first day after treatment. Nevertheless, these scores were not statistically significantly different after 3, 7, and 14 days of treatment between the two groups. Conclusions: Biodentine can be used as an apical plug to treat immature permanent molars with apical lesions in a single visit in children. Biodentine showed favorable outcomes in apical lesions healing, which was comparable to MTA but with a decreased treatment time associated with its use.

## 1. Introduction

Due to trauma or caries in immature teeth, dental pulp necrosis stops the formation of dentin and root growth; thus, the root canals remain wide with thin walls and open apices. Apexification aims either to induce closure of the apical foramen with calcified tissue or to create an artificial apical barrier in a root with an open apex [1]. Calcium hydroxide dressings have been used to induce calcified tissues formation at the root end in these roots. Although this material is effective with predictable results [2], it has several clinical drawbacks, such as the long time required for calcified tissues formation, the need for multiple visits and patient commitment, coronal leakage, and reinfection due to loss of temporary restoration between treatment sessions, susceptibility of the tooth to fracture, and the nature of formed barrier, which, although calcified, is porous and is sometimes even found to contain small amounts of soft tissue [3,4,5].

A few dental materials have been introduced to address some of these drawbacks. Mineral trioxide aggregate (MTA) cement, which has good sealing ability and boosts periradicular tissue regeneration, can be packed with a carrier, delivered into the apical third, injected as batches, and condensed vertically with a hand plugger to form apical plugs at the end of immature roots with or without periapical lesions [6]. After setting the apical plugs, the remaining part of the canal is obturated with gutta-percha and a sealer. This technique has multiple advantages, importantly a shorter treatment time and a good apical seal [1]. However, MTA has numerous shortcomings, including poor handling features, long setting time (3–4 h), the potential to discolor teeth, and high cost [6]. The extended setting time is a significant drawback of MTA use in children’s teeth. The treatment is preferred to be completed in a single session in patients requiring pharmacologic behavior management techniques, such as sedation or general anesthesia [7]. Biodentine was introduced in 2010 as bioactive calcium silicate-based cement and formulated using MTA-based cement technology, while claiming improvements of some of the MTA properties, such as physical qualities and handling. In addition, the setting time of Biodentine is about 12 min, providing a reasonable time for its application in a single-visit apexification procedure [8,9]. Reviewing several recent published studies and systematic reviews comparing MTA with Biodentine [10,11], no randomized controlled clinical studies were found comparing these two materials when used as an apexification cement in immature molars in children. Therefore, this study aimed to assess the clinical and radiographical healing of apical lesions of immature permanent molars in children after using Biodentine as an apical plug compared to MTA.

## 2. Materials and Methods

### 2.1. Ethical Approval and Protocol Registration

This study was approved by the Institutional Review Board (IRB) of Damascus University, Syria (ID # 1403, dated 9 March 2020) and it was registered on the Australian Newland Clinical Trials Registry (ID# ACTRN12621000493842).

### 2.2. Study Design and Settings

This study was conducted in the pediatric department, College of Dentistry, Damascus University from March 2020 and July 2021. This randomized clinical study utilized a two-arm parallel superiority design with a 1:1 allocation ratio. The apical plug was filled either with MTA or Biodentine.

### 2.3. Recruitment and Eligibility Criteria

The recruited participants involved children aged between 8 and 9 years with carious permanent mandibular first molars who attend the pediatric department during the study period. Patients were referred to the principal investigator if they had one or more molars with at least one open apex root (defined as root which root canal size equal or larger than #60 K-file) and presented with pulp necrosis and radiographic evidence of chronic apical periodontitis and periapical radiolucency greater than 3 mm. Children were excluded from the study if they had systemic diseases that compromised their general immune status, were classified as uncooperative (negative or definitely negative on the Frankl’s behavioral scale), or had unrestorable teeth. The parents of the children who agreed to participate in the study were given all the information regarding the study and signed informed consent prior to treatment.

### 2.4. Sample Size Calculation 

The sample size was calculated using G*Power software (Heinrich-Heine-Universität Düsseldorf, Düsseldorf, Germany) for the changes in the main outcome, the periapical index (PAI) score, in a previous study over 1 year of follow-up. The calculation used an equivalence limit of 0.5, standard deviation of 0.73, alpha level of 0.05, 85% power test, and two-tailed hypothesis. Furthermore, the sample size was increased by 15% to compensate for any drop-out. This resulted in 15 roots for each group.

### 2.5. Random Sequence Generation and Allocation Concealment

Teeth were assigned into MTA or Biodentine group using the simple randomization method, and a random sequence was created using the website www.random.org, accessed on 1 January 2022. 

The type of material chosen for apexification procedure was placed into opaque and sealed envelopes (15 envelopes per study group), and the patients were instructed to select any envelope randomly. Sequence generation and allocation concealment were performed by two different investigators not involved in the study intervention. 

### 2.6. Blinding 

As the current study was an interventional study, the treating clinician could not be blinded regarding the type of the material used to create the apical plug. However, the same clinician was not involved in the clinical and radiographic assessments after finishing the treatment and during subsequent follow-ups. The involved patients were not informed about their group assignment during the study period. The assessment of treatment outcomes was completed by two trained researchers (one PhD student and one MSc student) who were calibrated to the evaluation criteria and blinded to the type of the material used as an apical plug.

### 2.7. Clinical Procedures

Preoperative periapical radiographs of the included molars were taken using the paralleling technique with a film holder by a digital dental X-ray machine (Gendex GX, Lake Zurich, IL, USA) that was set at 60 kVp, 7 mA, and 0.32 s. 

**Figure 1:**
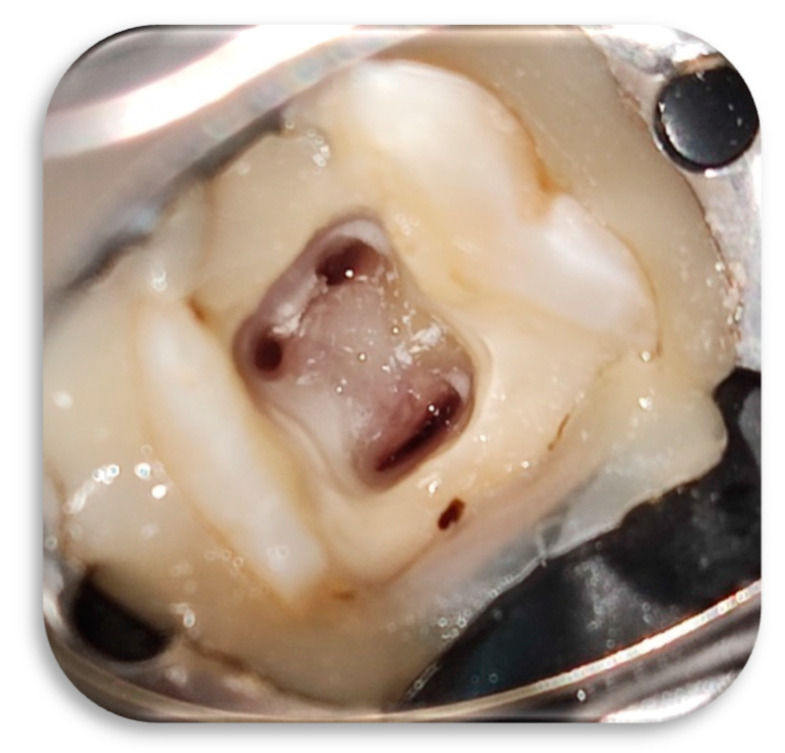
Pulp chamber after caries removed and access cavity refined.

**Figure 2:**
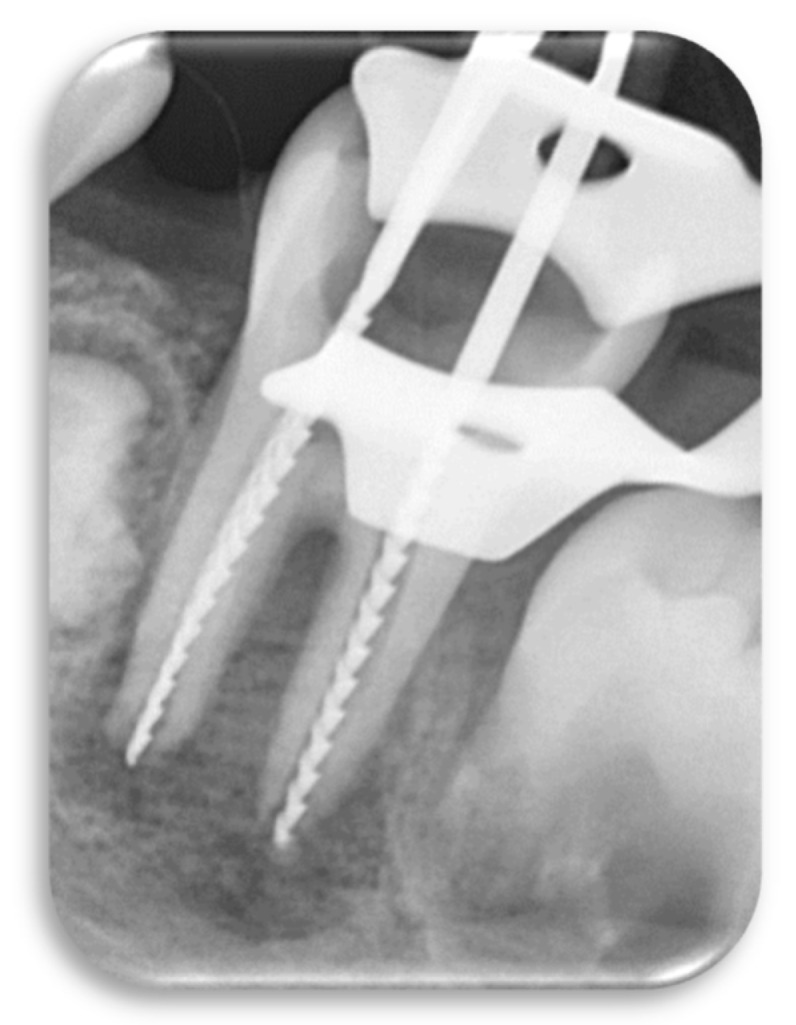
Determining the working length radiographically.

Under local anesthesia and rubber dam isolation, all caries were removed with a carbide fissure bur mounted on a low-speed handpiece. After penetrating into the pulp chamber, the access cavity was refined using an Endo-Z bur (Dentsply Maillefer, Tusla, OK, USA) mounted on a high-speed handpiece (Figure 1). The working length was determined radiographically using endodontic files and it was set at 1 mm shorter from the corresponding root apex (Figure 2).

### 2.8. Biomechanical Preparation

**Figure 3:**
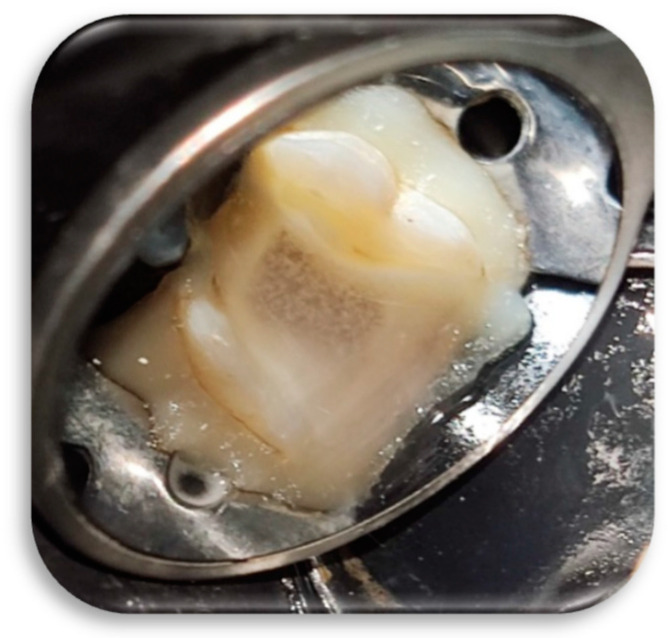
Pulp chamber after activation of hypochlorite in root canals.

Shaping and debridement the root canals was achieved by gentle instrumentation with Fanta AF3 rotary files (Fanta Dental Materials, Shanghai, China) consisting of two files: the SX file to expand the coronal third, and the #25/0.6 red file to expand the entire length of the canal. Each canal was irrigated with 5 mm^3^ of 1.3% sodium hypochlorite (NaOCI) (Merck, Darmstadt, Germany) using a 30-gauge endodontic irrigating needle (Sybron Endo, Crop, Orange, CA, USA) between the two files used, and then copiously irrigated (about 60 mm^3^ for each canal) with 1.3% NaOCI using the irrigating needle. As final irrigation, all canals were filled with NaOCl 1.3% and activated with a #40 U-file ultrasonic tip (Zipperer Co., Munchen, Germany) for 30 s for each canal (Figure 3). Subsequently, canals were irrigated with normal saline and filled up with Q-mix solution (Dentsply Tulsa Dental, Tulsa, OK, USA), which was activated with a #40 U-file ultrasonic tip for 30 s. Finally, canals were dried using sterile paper points (Gabadent, Guangdong, China).

### 2.9. Filling of the Root Canals

Canals with apical size less than #60 were filled using gutta-percha (Gabadent, Guangdong, China) and AH Plus sealer (Dentsply Sirona Endodontics) using the lateral condensation technique. The apical 4 mm of root canals with apical sizes greater than 60 were filled with either MTA (White ProRoot MTA, Dentsply, Tulsa, OK, USA) or Biodentine (Septodont, Saint-Maur-des-Fossés, France) plug while the rest of the canals were filled with gutta-percha and AH Plus sealer using the lateral condensation technique. 

In the MTA group, the material was inserted in the apical 4 mm of the canal using the MAP System (Produits Dentaires SA, Vevey, Switzerland) and adapted to the canal walls with a hand plugger (Dentsply, Tulsa, OK, USA). The correct placement and thickness of the apical plug were verified with a periapical radiograph. Then, a moist cotton pellet was placed in the canal to facilitate its setting, and the access cavity was temporarily restored with a glass-ionomer-based restoration named Medifil (Promedica Dental Material Company, GmbH, 24537 Neumunster, Germany). In the next day, under local anesthesia and rubber dam isolation, the temporary filling and the wet cotton pellet were removed. The setting of MTA was verified gently with a #40 k-file (Mani, INC. Company, Tochigi 321-3231, Japan). In the Biodentine group, the same procedure was followed except that the setting of the material was checked on the same visit after 12 min. 

### 2.10. Coronal Restoration

The pulp chamber was cleaned and filled with a glass-ionomer-based restoration (Fuji XI, GC Corporation, Tokyo, Japan), and the tooth was restored with a resin bonded restoration. To ensure sealing against coronal microleakage, a stainless-steel crown (3M ESPE, Dental Products, St. Paul, MN, USA) was inserted as a final restoration, and a postoperative radiograph was taken.

### 2.11. Clinical and Radiographic Evaluations 

Patients of both groups were recalled after 1, 3, 7, and 14 days of treatment, and the postoperative pain was recorded using a visual analog scale (VAS), where children set their pain levels by pointing (with a pen) along a 10 cm continuous line between two endpoints, ranging from the absence of pain to unbearable pain [12]. 

**Figure 4:**
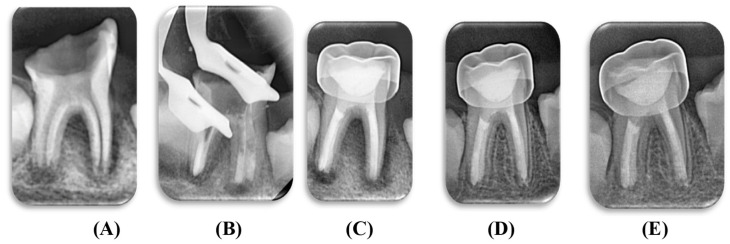
Apexification using MTA as an apical plug: (**A**) preoperative; (**B**) after placement of the apical plug; (**C**) postoperative; (**D**) 6 months follow-up; (**E**) 12 months follow-up.

**Figure 5:**
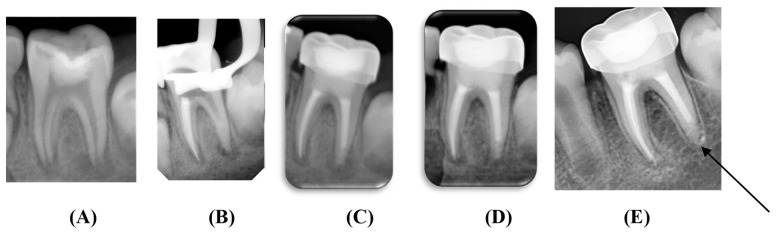
Apexification using Biodentine as an apical plug: (**A**) preoperative; (**B**) after placement of the apical plug; (**C**) postoperative; (**D**) 6 months follow-up; (**E**) 12 months follow-up. The arrow demonstrates the formation of apical barrier.

Follow-up radiographs were exposed using the same standardized settings at 6 and 12 months postoperatively (Figure 4 and Figure 5). The periapical status on the preoperative and follow-up radiographs was scored as follows according to the periapical index (PAI) system [13]: (1) normal periapical structures; (2) small changes in bone structure; (3) changes in bone structure with some mineral loss; (4) periodontitis with well-defined radiolucent area; (5) severe periodontitis with exacerbating features.

Lastly, the presence or absence of apical calcified barrier (ACB) at 12 months follow-up was recorded. The clinical and radiographic evaluations were performed by two blinded investigators who were calibrated to the above criteria. 

**Figure 6:**
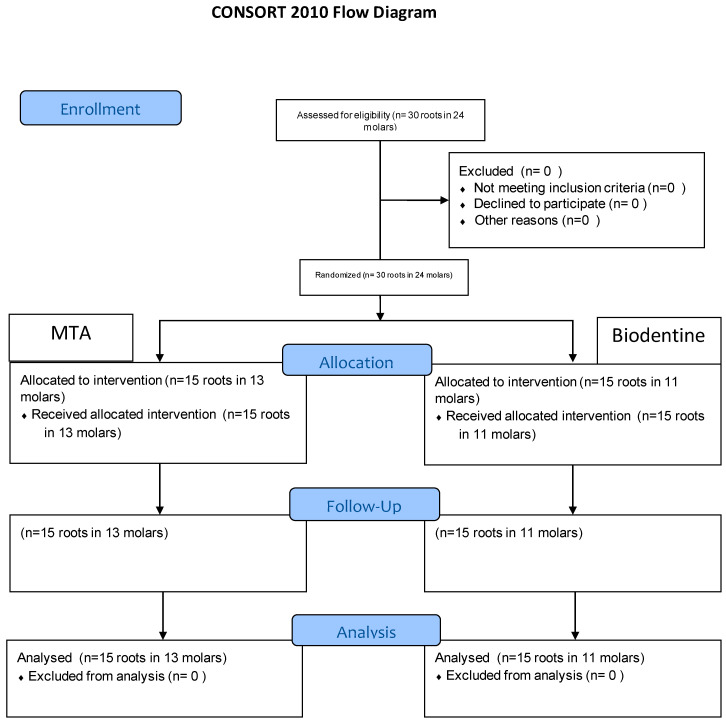
Flow chart for the study.

The flow of the patients in the study is described in Figure 6.

### 2.12. Statistical Analysis

The collected data were tabulated and analyzed using SPSS software (Version 20, IBM SPSS Inc., Chicago, IL, USA). The VAS scores between the groups at each timepoint were compared using the unpaired *t*-test; the PAI scores were compared using the Mann–Whitney U test, while the presence or absence of the ACB was compared using the chi-square test. In all comparisons, the level of significance was set at *p* < 0.05.

## 3. Results

A total of 24 mandibular first molars with 30 roots (15 per each group) were included in the study. No significant differences were reported between the groups regarding the age and gender of the children who were treated. 

Table 1 summarizes the clinical evaluation for postoperative pain between the two groups at each follow-up point. No significant differences in VAS scores were reported between the two groups except on first day after the final restoration was placed, where the Biodentine group had statistically higher pain scores than the MTA group (*p* = 0.043). All patients were also clinically asymptomatic 6 and 12 months after treatment. 

The results for changes in PAI scores between the two groups are summarized in Table 2. All roots showed progressive healing of the periapical lesions during follow-up intervals with no significant differences reported between the groups.

Formation of ABC was reported in four roots in the Biodentine group while it was not reported in the MTA group, and the chi-square test showed a significant difference between the two groups (*p* = 0.032).

## 4. Discussion

This clinical study is the first detailed study comparing the use of Biodentine as an apical plug-in single visit treatment to the MTA used across two visits in necrotic immature molars in children.

In this study, there were no statistically significant differences in the clinical and radiographic outcomes of immature mandibular first molars that received MTA or Biodentine apical plugs. No cases showed failure of treatment.

All root canals were prepared using the Fanta rotary system with minimal preparation to facilitate the access of instruments and irrigants to the apical region. The use of rotary files allowed shorter treatment time, enhancing patient cooperation, which is very important in pediatric dentistry [14]. 

A dilute concentration of sodium hypochlorite was used to perform repeated root canal irrigation to reduce the risk of hypochlorite leakage outside the open apex [1]. Ultrasonic activation with U-file tips was used to increase the effectiveness of disinfection and chemical dissolution of necrotic pulp tissues, as ultrasonic activation is considered safe for use in cases of open apices [15]. Q-mix was used as the final irrigant for 90 s. It effectively removes the smear layer because it contains EDTA, in addition to having an antibacterial effect and a prolonged action because it contains chlorhexidine [16]. It is also approved for use alone as final irrigant without sodium hypochlorite to maintain the best long-term effect against *Enterococcus faecalis* [17] Removal of necrotic pulp tissue remnants along with canal disinfection of the root canal system are required for successful apexification treatment. Both materials used in this study as apical plugs showed similar attachment of osteoblast cells on their surfaces [18] and significant antimicrobial effect against *E. faecalis* [19]. In addition, they showed good marginal adaptation and similar sealing ability [20,21]. The thickness of the apical plug plays an important role in maintaining an adequate apical seal. Abbas et al. compared the bacterial leakage of MTA and Biodentine when used as apical plugs of 2- and 4-mm thickness [22]. The result showed that the 4 mm apical plug of Biodentine showed the least amount of bacterial leakage, followed by 2 mm MTA and 4 mm MTA, while the 2 mm apical plug of Biodentine showed the maximum bacterial leakage. Therefore, in the current study, a 4 mm thickness plug of both materials was used to ensure an adequate apical seal [22].

Overall, the postoperative pain level was similar between the two groups except at the first day postoperatively, where the Biodentine group showed a significantly higher pain score than the MTA group. The long setting reaction of MTA needs moisture [23] as it is more likely to take it from the inflammatory filtrate of the apical lesion, and it also releases greater amounts of calcium ions during the first hours of hardening compared to Biodentine [24], which contributes to modifying the acidity of the apical lesion, thus decreasing pain on the first day. Furthermore, the single-visit approach using Biodentine causes direct occlusal loading when using the stainless-steel crown, which leads to increased pressure on the periodontal ligament, thus increasing pain on the first day. 

In the current study, the formation of a calcified barrier was noted in four cases of the Biodentine group, while it was absent in the MTA group. Biodentine showed a greater ability to produce apatite crystals and release dental elements than MTA [25], and it provided an appropriate environment for osteoblast and periodontal ligament or PDL cell growth [26]; this may explain the appearance of a calcified barrier under four Biodentine apical plugs after 12 months of treatment.

Biodentine promoted greater survival and differentiation of stem cells of the apical papilla (SCAP) and an increase in the odontoblastic marker DSPP, promoting the sealing of open-apexed root canals and peri-apical healing. MTA appeared to promote greater osteoblastic differentiation according to a greater expression of the osteoblastic marker Integrin Binding Sialoprtein or IBSP [27]. It seems that these two modes of healing are radiologically similar. Thus, Biodentine and MTA can be considered for the treatment of immature teeth in children. 

Khalaf [28] and Yadav et al. [29] used hand files and 0.5% sodium hypochlorite to debride and prepare necrotic immature teeth and compared MTA and Biodentine in apexification; they concluded that both MTA and Biodentine can be used successfully in apexification procedures for immature nonvital permanent teeth with a high rate of success. Our current study corroborates this finding in children. 

In this study, it was a challenge to standardize the radiographic images in the follow-up periods because the children were in the mixed occlusion period, and the occlusal level was not stable as a guide for the radiographic sensor holder. In addition, the size of the sensor along with its holder was unacceptable for some children who had severe vomiting or gagging reflex. Further studies with a larger sample size are warranted to confirm this study’s findings, as it was not easy to collect a larger number of participating children in this research due to the spread of COVID-19 during the period of conducting the study and follow-up. 

## 5. Conclusions

The results of this research indicate that following the proposed protocol of treatment led to recovery in all cases of the research. Biodentine had similar results in terms of apical lesion healing and clinical recovery compared to MTA; hence, it can be used as an apical plug to treat immature permanent molars with apical lesions in a single visit, especially in uncooperative children, as well as enable treatment under general anesthesia or deep sedation because of the shorter treatment time provided.

## Figures and Tables

**Table 1:** Comparison of postoperative pain (measured with VAS scale) between the two groups.

Follow-up	Biodentine		MTA		*t*-Value	*p*-Value
	Mean ± SD	Range	Mean ± SD	Range		
Baseline	2.47 ± 1.88	0–7	1.73 ± 1.16	0–3	1.282	0.210
1 day	4.27 ± 1.53	2–7	3.20 ± 1.21	1–5	2.117	0.043 *
3 days	2.73 ± 1.67	1–5	2.20 ± 1.57	0–5	0.903	0.374
1 week	1.80 ± 1.61	0–4	0.93 ± 1.22	0–3	1.659	0.108
2 weeks	1.13 ± 1.55	0–4	0.40 ± 0.74	0–2	1.653	0.110

* Statistically significant at *p* < 0.05.

**Table 2:** Comparison of PAI scores between the two groups.

Follow-up Period	Plug Material	PAI Score						U-Value	*p*-Value
		1	2	3	4	5	Mean Rank		
Baseline	Biodentine	0	0	0	14	1	4.63	105	0.317
	MTA	0	0	0	15	0	4.77		
6 months	Biodentine	4	9	2	0	0	1.90	106	0.753
	MTA	3	10	2	0	0	2.13		
12 months	Biodentine	9	6	0	0	0	1.30	90	0.240
	MTA	12	3	0	0	0	1.23		

## Data Availability

Deidentified data are available upon written request to the corresponding author.
